# Semaphorin3A induces nerve regeneration in the adult cornea-a switch from its repulsive role in development

**DOI:** 10.1371/journal.pone.0191962

**Published:** 2018-01-25

**Authors:** Min Zhang, Qiang Zhou, Yuncin Luo, Tara Nguyen, Mark I. Rosenblatt, Victor H. Guaiquil

**Affiliations:** Department of Ophthalmology and Visual Sciences, University of Illinois-Chicago, Chicago, Illinois, United States of America; Szegedi Tudomanyegyetem, HUNGARY

## Abstract

The peripheral sensory nerves that innervate the cornea can be easily damaged by trauma, surgery, infection or diabetes. Several growth factors and axon guidance molecules, such as Semaphorin3A (Sema3A) are upregulated upon cornea injury. Nerves can regenerate after injury but do not recover their original density and patterning. Sema3A is a well known axon guidance and growth cone repellent protein during development, however its role in adult cornea nerve regeneration remains undetermined. Here we investigated the neuro-regenerative potential of Sema3A on adult peripheral nervous system neurons such as those that innervate the cornea. First, we examined the gene expression profile of the Semaphorin class 3 family members and found that all are expressed in the cornea. However, upon cornea injury there is a fast increase in Sema3A expression. We then corroborated that Sema3A totally abolished the growth promoting effect of nerve growth factor (NGF) on embryonic neurons and observed signs of growth cone collapse and axonal retraction after 30 min of Sema3A addition. However, in adult isolated trigeminal ganglia or dorsal root ganglia neurons, Sema3A did not inhibited the NGF-induced neuronal growth. Furthermore, adult neurons treated with Sema3A alone produced similar neuronal growth to cells treated with NGF and the length of the neurites and branching was comparable between both treatments. These effects were replicated in vivo, where thy1-YFP neurofluorescent mice subjected to cornea epithelium debridement and receiving intrastromal pellet implantation containing Sema3A showed increased corneal nerve regeneration than those receiving pellets with vehicle. In adult PNS neurons, Sema3A is a potent inducer of neuronal growth in vitro and cornea nerve regeneration in vivo. Our data indicates a functional switch for the role of Sema3A in PNS neurons where the well-described repulsive role during development changes to a growth promoting effect during adulthood. The high expression of Sema3A in the normal and injured adult corneas could be related to its role as a growth factor.

## Introduction

The cornea is the most densely innervated tissue in the human body. Corneal nerves respond to multiple sensation modalities and also provide trophic function to maintain a healthy and clear epithelium [1]. Corneal nerves can be damaged by a myriad of conditions such as trauma, infection, surgery, diabetes and chemical burns [2, 3]. These injuries may lead to changes in neuronal innervation, resulting in altered functional sensation, and to sequelae such as dry eye syndrome [4].

Growth factors play an important role in the maintenance and renewal of normal corneal epithelium and nerve endings. They also have a vital role in corneal wound healing, mediating the proliferation of epithelial and stromal tissue, the regeneration of sub basal nerves and affecting the remodeling of the extracellular matrix (ECM). These functions depend on a complex interplay between growth factors of different types, the ECM, and regulatory mechanisms of the affected cells that may limit or enhance their potency [5, 6].

Semaphorins are a large family of membrane bound and of secreted glycoproteins that were initially described as axon guidance proteins with a role during neuronal pathfinding and repulsive effects on growing axonal growth cones during development [7]. Semaphorin3A (Sema3A) belongs to the Semaphorin Class 3 secreted proteins and has been described as an axon guidance and growth cone repellent protein during development [8–10]. After birth and into adulthood the role of Sema3A is more pleotropic and has been associated with nerve pathfinding [11], dendritic growth in newborn hippocampal neurons [12], inhibition of sympathetic and parasympathetic neurons [13], and several other effects distinct from its originally described neuronal growth repulsion [11, 14]. In the eye Sema3A is highly expressed in the chicken lens during development, and its inhibitory effects on nerve growth are essential for the proper innervation of the cornea [15, 16]. In the murine adult cornea Sema3A is abundantly expressed in the superficial epithelium and becomes highly upregulated upon injury [17, 18]. However, this high expression level of Sema3A and other members of the Semaphorin family in the cornea [19], clearly does not translate to an inhibition of nerve regeneration, since the neuroregenerative potential of injured neurons remains high and can be enhanced by addition of growth factors such as NGF, VEGF-A or VEGF-B [5, 10, 20, 21]. The role of Sem3A on injured adult PNS derived neurons, such as those that innervate the cornea is largely unknown, and it is possible that they may play a different role from that observed in embryos. The main goal of the present study was to determine the role of Sema3A in adult PNS neurons derived from the trigeminal ganglia (TG) and dorsal root ganglia (DRG) and to establish whether the high level of expression in the cornea correlates with neuronal growth required during injuries. We compared the inhibitory role of Sema3A in vitro in embryonic neurons to adult neurons and performed in vivo cornea nerve injuries to evaluate the effects of Sema3A addition on nerve regeneration.

## Materials and methods

### Animals

All experiments were performed according to the guidelines of the Association for Research in Vision and Ophthalmology Statement for the Use of Animals in Ophthalmic and Vision Research. The neurofluorescent thy1-YFP mice were purchased from The Jackson Laboratories (Bar Harbor, ME) and bred as described [22]. For in vivo experiments, mice were anesthetized with intraperitoneal injections of a combination of ketamine (20 mg/kg; Phoenix Scientific, St. Joseph, MO) and xylazine (6 mg/kg; Phoenix Scientific). For terminal experiments, mice were sacrificed by CO_2_ inhalation followed by cervical dislocation.

### Trigeminal neuronal and corneal epithelial cell cultures

Trigeminal ganglia (TG) were removed from thy1-YFP mice and cultured as previously described [20, 23]. In brief, ophthalmic branches of the trigeminal nerves were harvested and cleaned from contaminating tissue, then subjected to enzymatic digestion with papain and collagenase/dispase (Worthington Biochemicals, Lakewood, NJ) and separated in Percoll gradients by centrifugation. Cell cultures were maintained in 96-well plates coated with 20 μg/mL laminin (Sigma, St. Louis, MO) and supported with media (Neurobasal; Invitrogen, Carlsbad, CA) supplemented with 2% fetal bovine serum (FBS) and 1% antibiotic/antimycotic (ABAM; Gibco, Grand Island, NY). Mouse corneal epithelial sheets were isolated using dispase treatment, as described by Kawakita et al.[24]. Epithelial sheets were rendered into single cells by 0.25% trypsin-EDTA (Gibco). Dissociated cells were seeded in six-well plates with corneal epithelium growth medium (Epilife; Cascade Biologics, Portland OR) with 20 μM calcium, 1% human corneal growth supplement (Cascade Biologics), 0.01 μg/mL mouse epidermal growth factor (Sigma), 0.1 nM cholera toxin A subunit from *Vibrio cholerae* (Sigma), and 1% ABAM (Gibco) until they were 90% confluent.

### Cornea epithelium injury

Mice were anesthetized with a mix of ketamine/xylazine and 1 drop of 0.5% proparacaine hydrochloride ophthalmic solution (Bausch & Lomb, Tampa FL) was applied to the eye to deliver local corneal anesthesia before injury. A 2-mm circular area of corneal epithelium was demarcated using a trephine and was gently removed and collected using a scalpel blade without damaging the underlying stroma. Mice were allowed to recover, and the corneal epithelium was collected again at 1, 3, 5 and 15 days post injury for gene expression analysis as detailed below.

### Class 3 semaphorins gene expression in cornea epithelium and trigeminal ganglia

Total RNA was isolated from mouse cultured corneal epithelial cells (passage 0), cultured trigeminal neurons (passage 0), cornea epithelial tissue and trigeminal ganglion tissue according to the manufacturer’s protocol (RNeasy Mini Kit and RNeasy Micro Kit; Qiagen, Germantown, MD). Whole mouse embryos (embryonic day [E] 11.5) were used as a positive control to ensure finding detectable levels of class 3 Semaphorin gene expression. RNase-free DNaseI was used to remove DNA. RNA was stored at -80°C in the presence of RNase inhibitor (Invitrogen, Carlsbad, CA), and RNA concentration and quality were assessed (2100 Bioanalyzer; Agilent, Santa Clara, CA). cDNA was generated according to the manufacturer’s protocols (Superscript III First-Strand Synthesis System; Invitrogen). Total RNA (500–1000 ng) was reverse transcribed (Oligo(dT)20; Invitrogen). Of the resultant cDNA, 50 ng was used for each PCR amplification in accordance with the manufacturer’s protocol (MasterTaq Kit; Eppendorf, Hamburg, Germany). Specific primers [22, 25] were designed as follows: SEMA3A forward (5’-GTTGTAGACCGGGTGGATGC-3’) and reverse (5’-TCGGAGCAGTGAGTCAGTGG-3’), SEMA3B forward (5’-GAGGACTCTGCCGCTATCAC-3’) and reverse (5’-CTCCACACCCAACACCTTCT-3’), SEMA3C forward (5’-CACCCAATATGAGAACCAA-3’) and reverse (5’-TCGCCAAGCAGCAGTCC-3’), SEMA3D forward (5’-AGCACCGACCTTCAAGAGAA-3’) and reverse (5’-GTGCATATCTGGAGCAAGCA-3’), SEMA3E forward (5’-TCAAGTATCCGGGAAGCCTTTA-3’) and reverse (5’-ACATGTCGCAGTGATGGAATCT-3’), SEMA3F forward (5’-AGGTGGATGCAGCTGATGG-3’) and reverse (5’-GGAATTGAAACCACGGCACT-3’) and GAPDH control forward (5’-ACCACAGTCCATGCCATCAC-3’) and reverse (5’-TCCACCACCCTGTTGCTGTA-3’).

Amplifications were performed using the following cycling parameters: 94°C for 3 minutes, followed by 30 cycles of 94°C for 45 seconds, annealing temperature (Sema3A, 65°C; Sema3B, 52°C; Sema3C, 60°C; Sema3D, 52°C; Sema3E, 58°C; Sema3F, 60°C; GAPDH, 60°C) for 30 seconds and 72°C for 45 seconds, and a final extension cycle of 72°C for 5 minutes [25]. Glutaraldehyde-3- phosphate dehydrogenase (GAPDH) was the loading control. A negative control without cDNA was also performed and PCR products were resolved in 1.5% agarose gels. The corneal epithelium gene expression changes for Sema3A and Sema3F after debridement were evaluated by quantitative PCR, using predesigned mouse Sema3A (catalog number 4331182, assay ID Hs00173810_m1), Sema3F (4351372, assay ID Hs01030910_g1) and GAPDH (catalog number 4331182, assay ID Mm99999915_g1) TaqMan gene expression assays and TaqMan Gene Expression Master Mix in the StepOnePlus Real-Time PCR system (Applied Biosystems, Carlsbad, CA). Samples were assayed in triplicate in a 20 μl reaction volume according to the manufacture’s protocol. GAPDH was evaluated as an internal control to normalize Sema3A and Sema3F expression. Negative controls without cDNA or RT were included.

### Trigeminal ganglia neuronal growth assay

Trigeminal ganglion cells were obtained from 3–8 weeks old thy-YFP mice and cultured as described previously [20, 23]. In brief, ophthalmic branches of the trigeminal ganglia were harvested from thy1-YFP mice. Tissues were subjected to enzymatic digestion with papain and collagenase/dispase (Worthington Biochemicals, Lakewood, NJ). Trigeminal neurons were separated in Percoll gradients by centrifugation at 1300 g for 10 min and seeded in poly-D-lysine-coated plates containing Neurobasal A medium supplemented with 1% B27 and 1% penicillin/streptomycin (Gibco, Grand Island, NY). To determine the induction of neuronal growth we used as a positive control nerve growth factor (NGF), a well-known inducer [10, 26] and evaluated the effects of Sema3A addition to the cultures. TG neuronal cells were either treated with growth medium only as negative control or treated with 10–200 ng/ml murine NGF (Cat number N60009, Sigma, St. Louis, MO) and/or recombinant human Sema3A (Catalog number 1250-S3, R&D Systems, Minneapolis, MN). Additionally, some cultures were treated with recombinant mouse or human Sema3A from Sino Biological Inc. (Catalog numbers 50631-M01H and 10758-H01H respectively, Beijing, China). The treatments were replenished every other day. Neurite formation and growth was followed up to 7 days. Neuronal growth was established as those cells that presented neurite extension that was 2 fold over the diameter of the cell body. The neurite elongation was classified into three types: (i) short (neurites between 50 and 100 μm in length), (ii) medium (neurites between 150 and 300 μm in length), and (iii) long (neurites longer than 400 μm). Neurons were counted to obtain the percentage of neurons with neuronal growth and the total neurons per dish. Images were obtained using an AxioObserver fluorescent microscope (Carl Zeiss Microimaging GmbH, Jena, Germany) and processed with Photoshop CS5.1 to generate images compatible to use with Neuron J software (a Image J plugin available at https://imagescience.org/meijering/software/neuronj) to trace neuronal length and branching.

### Dorsal root ganglia neuronal growth assay

Dorsal root ganglia from E15 embryos or adult thy1-YFP mice (3–8 weeks old) were obtained as described [27]. Briefly, the spinal column was dissected out from the base of the skull to the level of the femurs, then it was cut down the mid-line and the spinal cord and meninges removed, unwanted axons were removed before extracting the DRG. The ganglia were subjected to enzymatic digestion with papain for 20 min at 37°C followed by collagenase/dispase digestion for 20 min at 37°C (Worthington Biochemicals, Lakewood, NJ). DRG neurons were centrifuged at 200g for 2 min, incubated in culture medium containing Neurobasal A medium supplemented with 1% B27 and 1% penicillin/streptomycin (Gibco, Grand Island, NY) and seeded on dishes coated with laminin/poly-D-lysine. The neurons were incubated for 2 h and then treated with either growth medium alone as negative control or with NGF, Sema3A or combination in a dose dependent manner as described above. The embryonic neurons were incubated with NGF to induced neurite growth. NGF was carefully replenished every other day to avoid disturbing the attached neurons. For this one third medium volume was removed and replaced it with same volume fresh medium containing NGF. At day 3 neurons either continued with NGF treatment or Sema3A was added to the dish without suspending the NGF treatment. Neuronal growth was established as for TG neurons described above. The effect of these treatments on neurons was recorded by images taken before and every 15 min after the addition of Sema3A using 16h time-lapse imaging. The acquisition of images and analysis were performed as described above.

### Corneal intrastromal micropocket assay

Anesthetized 6–8 weeks old thy1-YFP mice were subjected to a 2mm central corneal epithelium debridement [20], immediately followed by implantation of a sucralfate/hydron pellet impregnated with human or mouse recombinant Sema3A (100 ng/pellet, Sino Biological Inc. catalog number 50631-M01H, Beijing, China) or vehicle (PBS) into a corneal micropocket made by a partial lamellar incision parallel to the limbus [28, 29]. The corneas were monitored for signs of infection, inflammation or neovascularization and harvested 7 days post procedure. The excised corneas were processed for cryosections or flat mounted as described below for visualization of Sema3A expression and cornea nerve regeneration using immunofluorescence staining.

### Evaluation of Sema3A expression in corneal cryosections

Whole eyes from intact mice (basal control), mice subjected to corneal epithelium debridement only or mice subjected to corneal epithelium debridement and pellet implantation (vehicle or Sema3A) were collected after 1, 5 and 7 days post injury. Eyes were washed twice in PBS and immediately embedded in OCT compound (Tissue-Tek, Sakura, Torrance, CA) and frozen at -80°C until sectioning. Eye sections of 10 μm thickness were obtained by using a Cryostar NK50 (ThermoFisher, Kalamazoo, MI), dried at room temperature and store at -20°C. For immunofluorescence staining, section were fixed in ice cold methanol for 5 min, washed 3 times in PBS, blocked (2% BSA, 2.5% donkey serum in PBS) for 1h at room temperature and incubated overnight with 1:200 dilution of rabbit anti Sema3A antibody (cat # 21925, Proteintech, Rosemont, IL) at 4°C. Sections were washed 3 times in PBS and incubated at room temperature for 1 hour with donkey anti rabbit Cy3 secondary antibody at 1:500 dilution (Jackson Immunoresearch Laboratories, West Grove, PA). Sections were washed 3 times with PBS, air dried and mounted in Vectashield mounting medium with DAPI (Vector labs, Burlingame, CA). Sections were imaged using a fluorescent microscope as described above.

### Evaluation of Sema3A expression and nerve regeneration in corneal whole mounts

Harvested corneas from thy1-YFP mice were fixed for 10 min (for Sema3A staining) and 30 min (for B3 tubulin staining) in cold acetone, washed 3 times for 5min in PBS and then blocked and permeabilized in buffer containing 1% BSA, 0.25% Triton X-100 (EMD Chemicals, Darmstadt, Germany), and 2.5% donkey serum (Abcam, Cambridge, MA) in PBS for 1 h. Corneas were incubated with either 1:200 dilution of rabbit anti Sema3A or 1:400 dilution of rabbit anti-β3 tubulin antibody (catalog number 18207 Abcam, Cambridge, MA) overnight at 4°C, followed by incubation with 1:500 donkey anti rabbit Cy3 or 1:400 dilution of AlexaFluor568-conjugated goat anti-rabbit IgG (Catalog number A11011 Life Technologies, Carlsbad, CA) respectively, at room temperature for 1 hr and flat mounted in Vectashield containing DAPI. Corneas were imaged using an AxioObserver fluorescent microscope (Carl Zeiss Microimaging GmbH, Jena, Germany). Sema3A expression was analyzed using 10x and 20x images obtained using phase and fluorescence to show the pellet location and corneal epithelium staining. Nerve regeneration was analyzed on mosaic images obtained at 20x magnification. Images were focused on the superficial nerves and cover the entire cornea epithelium debrided area. Images were converted to TIF files and imported into nerve tracing software Neurolucida 9 (MBF Bioscience, Williston, VT, USA). Nerves were traced using the semi-automatic module and those missing nerve fibers were manually added for quantification. Nerve regeneration is expressed as the total length of superficial nerve fibers that were present in the whole corneal debrided area. Experiments were repeated 3 times using five animals per experiment.

### Statistics

Data are presented as mean ± SD. The significance of differences was evaluated using either t-test or analysis of variance (ANOVA), with P < 0.05 considered statistically significant unless otherwise stated.

## Results

### Semaphorins are highly expressed in normal and injured cornea

**Fig 1:**
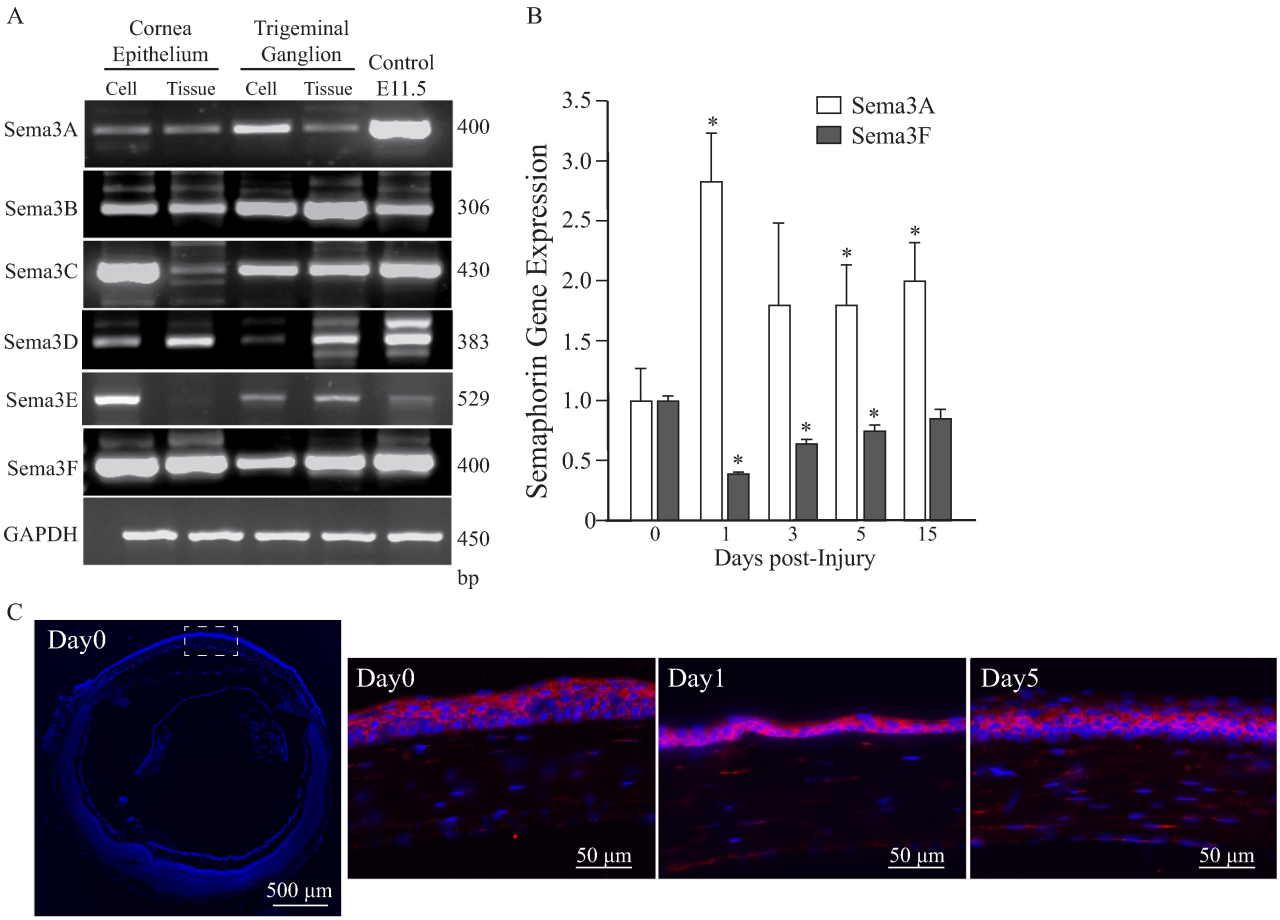
Expression of class 3 Semaphorin family members in mouse cornea and trigeminal ganglia. RT-PCR and qPCR of class 3 Semaphorins was performed in isolated cells and tissues as detailed in Materials and Methods. Immunofluorescence staining was performed in eyes from intact mice (basal control) and mice subjected to corneal epithelium debridement. **(A)** All class 3 Semaphorins are expressed in the corneal epithelium and TG, with Sema3B and Sema3F showing higher and consistent levels. **(B)** The gene expression of Sema3A and Sema3F after corneal epithelium debridement significantly changed from their basal level. While Sema3F expression in the corneal epithelium is reduced to 50% after one day post debridement and slowly recovers to normal levels after 2 weeks, Sema3A is sharply upregulated soon after injury and remains elevated over the entire evaluated period. Values represent mean ± SD, experiments were performed in triplicate (for each experiment tissues were collected from n = 4 mice, * indicates p< 0.05 vs day 0). (**C**) Eye crossections stained with anti Sema3A (red) antibody and DAPI (blue, nuclear staining) shows apical expression of Sema3A in basal control. Upon injury there is high expression of Sema3A in the basal layers of the epithelium. Dashed white-square in left image shows the area of analysis. Images are representative of n = 4 mice.

In order to characterize the gene expression of Class 3 Semaphorins in the mouse cornea epithelium and trigeminal ganglion cells and tissues, semi quantitative RT-PCR and qPCR were used (Fig 1). The TG neurons isolated from neurofluorescent thy1-YFP mouse can easily be identified by fluorescence and used to establish the purity of isolated cell preparation. We found that all the Sema3 family members are expressed in both cultured cells and tissues with Sema3B and Sema3F more abundantly expressed (Fig 1A and S1 Fig). After corneal injury by epithelial debridement, we found significant differences on the injury-response pattern of Sema3F and Sema3A expression in the cornea epithelium. The quantitative analysis of gene expression showed that Sema3F was reduced to 50% at day 1 post debridement and slowly recovered to normal level after 14 days. On the contrary, the expression level of Sema3A quickly increased 3 fold over baseline after day 1 and remained elevated 2 fold after 14 days (Fig 1B). The gene expression of others class 3 Semaphorins was not significantly altered. Additionally, we analyzed the expression of Sema3A by immunofluorescence staining (Fig 1C). We found high expression of Sema3A in both intact and injured corneas. In the uninjured state Sema3A is preferentially located in the outermost layers of the corneal epithelium, upon injured there is high expression of Sema3A in the basal layer of the regenerating epithelium and becomes more intermediately located after 5 days post-debridement.

### Sema3A induced axonal retraction and growth cone collapse in embryonic but not in adult DRG neurons

**Fig 2:**
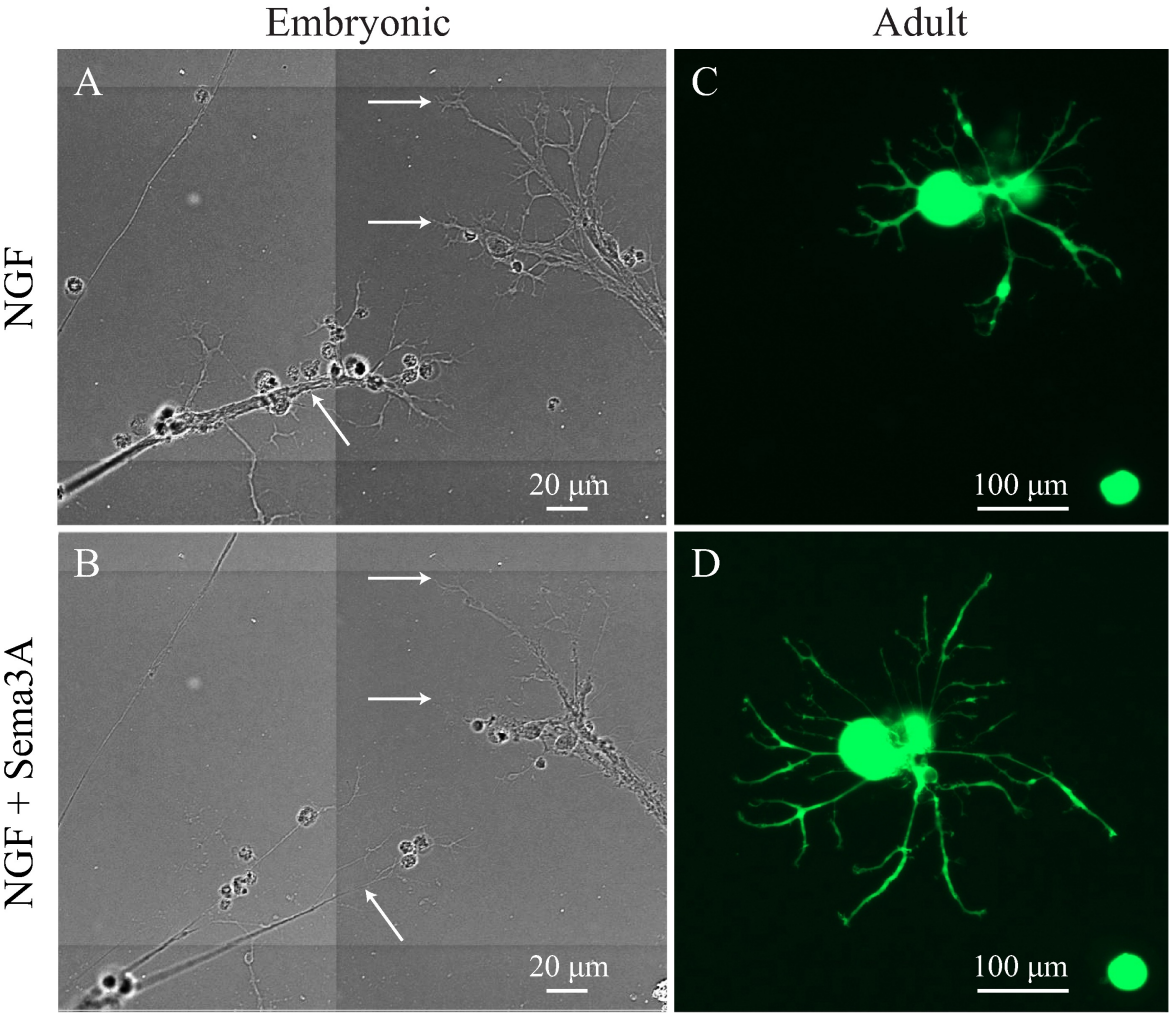
Sema3A induced axonal cone retraction and collapse in embryonic but not adult DRG neurons. Isolated embryonic and adult DRG were treated with 50 ng/ml NGF to induce neuronal growth and then Sema3A was added to the cultures to test the inhibitory effect using time lapse imaging analysis. (**A)** In embryonic DRG, NGF induced a fast growth of neurites that developed long axons with extensive branching and a clear growth cone area (white arrows). **(B)** After 3 days in culture, Sema3A was added and time-lapse imaged recorded. Addition of Sema3A induced fast growth cone collapse and axonal retraction (white arrows) here shown after 10 h. (**C**) In adult DRG neurons, NGF induced extension and branching of neurites. **(D)** Addition of Sema3A has no inhibitory effect on the neurite growth and no regression or collapse of axons was observed. Representative images of neurons observed in experiments performed in triplicate, each dish with an average of 200 neurons; all neurons in every dish were evaluated (scale bars A and B = 20 μm, C and D = 100 μm).

Since Sema3A was highly expressed after corneal injury, we investigated if it may have any effects on neuronal growth. For this, we isolated DRG derived neurons from thy1-YFP E15 embryos, which contain fewer fibroblasts and Schwann cells, making it easier to obtain purified neurons [30]. Neurons were incubated with 50 ng/ml NGF to induce neurite growth, and were supplemented with fresh NGF every other day. When long neurites with large growth cones were formed, usually at day 3, cells were transferred to the microscope for time lapse imaging recording. For this cells were incubated at 37 °C on a humidified incubation chamber containing 5% CO_2_, using the incubator module of the AxioObserver microscope. Four hours after imaging began, 50 ng/ml NGF or Sema3A was added to the culture and remained in the culture as the time-lapse imaging continued overnight. Addition of extra NGF did not induce any immediate changes in the morphology and branching of the neurites (Fig 2A). However, addition of Sema3A to embryonic DRG cultures quickly induced axonal growth cone collapse and axon retraction, which was observed by video recording as early as 30 min post addition and continued until complete regression of the axons, which retracted at a speed of 30–40 μm/h. Fig 2B and S3 Fig (video), shows the extensive axonal regression and collapse of the growth cone after 10 h incubation with Sema3A. In a separate experiment, adult DRG neurons derived from thy1-YFP mouse were incubated with 50 ng/ml NGF to induce neuronal growth and imaged after 2 days in culture (Fig 2C). After imaging began, 50 ng/ml Sema3A was added to the dishes and neurons incubated for 24 h. Neurons imaged after these treatments showed no signs of axonal cone growth collapse or retraction of neurites (Fig 2D) and neuronal growth was similar to that observed in control cells treated with only NGF. Wide phase mosaic images of embryonic and uncropped adult neurons are shown on S2 Fig.

### Sema3A does not affect NGF induced neuronal growth in adult DRG or TG neurons

**Fig 3:**
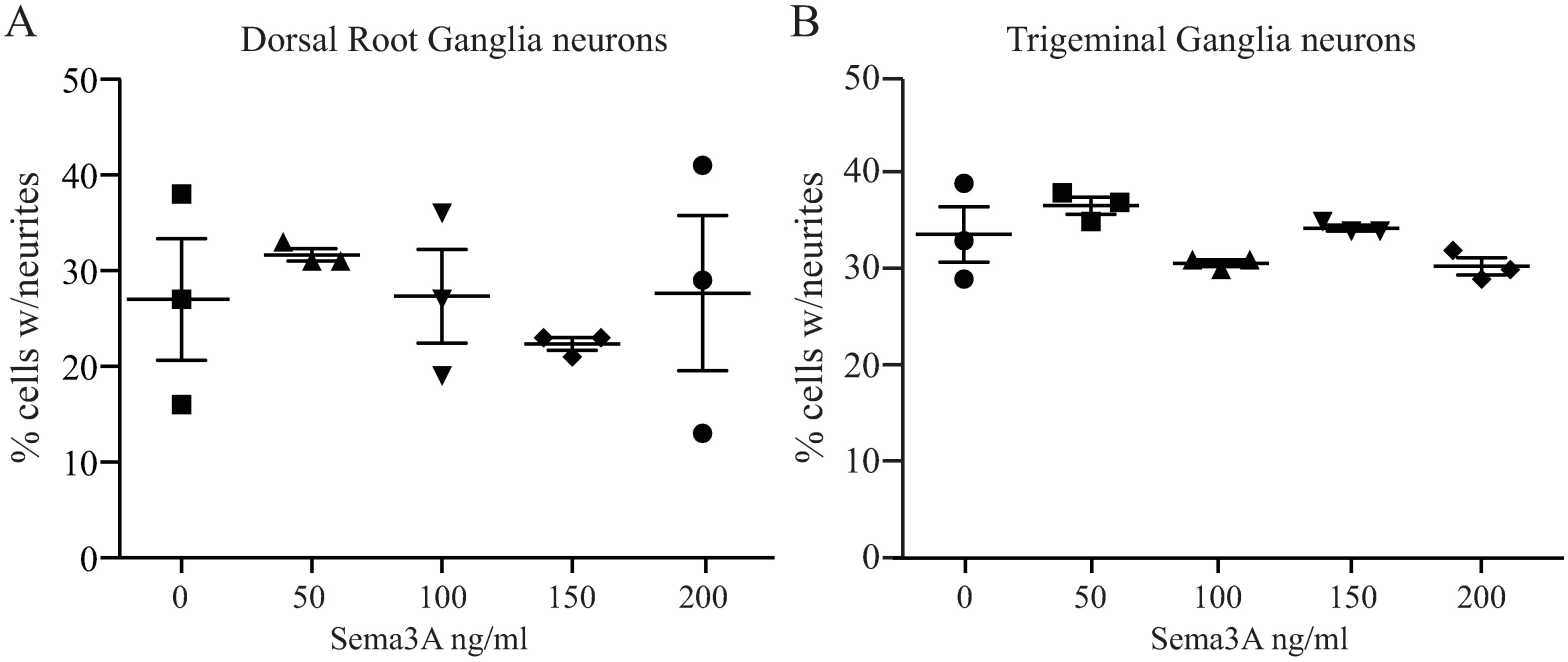
Sema3A does not inhibit the NGF induced neuronal growth of adult DRG and TG neurons. Isolated DRG and TG neurons were incubated with NGF for 3 and 2 days respectively to induce neuronal growth and then treated with different concentrations of Sema3A to determine any significant growth inhibitory effect on these PNS neurons. Neurite growth was evaluated 24 h after addition of Sema3A. **(A)** We found that about one third of the isolated neurons responded to the NGF treatment and addition of Sema3A produced no inhibitory effects such as the neurons kept growing similarly as compared to control cells treated with NGF alone. (**B**) TG neurons responded to NGF similarly to DRG neurons and addition of Sema3A did not alter the growth of neurites and no axonal retraction was observed. Neuronal growth was similar to controls that were resupplied with NGF over the course of the experiment. Values represent mean ± SEM, experiments were performed in triplicate, each dish had an average of 200 neurons for DRG and 60 neurons for TG; all neurons in every dish were evaluated.

Since we observed no inhibition of neuronal growth by Sema3A on NGF treated adult DRG neurons, we decided to determine if higher concentrations of Sema3A will block or induce arrest of NGF induced neuronal growth. For these experiments, cells were treated with 50 ng/ml NGF which induce potent neurite growth, and then with increasing concentrations of Sema3A. DRG neurons were incubated with NGF for 3 days and neuronal growth evaluated after overnight incubation with Sema3A at day 4. We observed no neurite growth inhibition at any doses of Sema3A tested and the percentage of cells that presented neurite extension was similar to cells treated with NGF alone (Fig 3A). Additionally, we tested the effects of Sema3A on isolated neurons derived from the TG that innervate the cornea, which constitute another model of PNS neurons. For these experiments, TG neurons were treated with NGF as above for 2 days and neuronal growth evaluated after overnight incubation with Sema3A at day 3. Sema3A did not alter the NGF induced neuronal growth in TG cells at any of the doses used. We did not observe changes in the percentage of neurons that developed neurites and no neurite retraction were observed (Fig 3B).

### Sema3A Acts as a growth factor and is a potent inducer of neuronal growth

**Fig 4:**
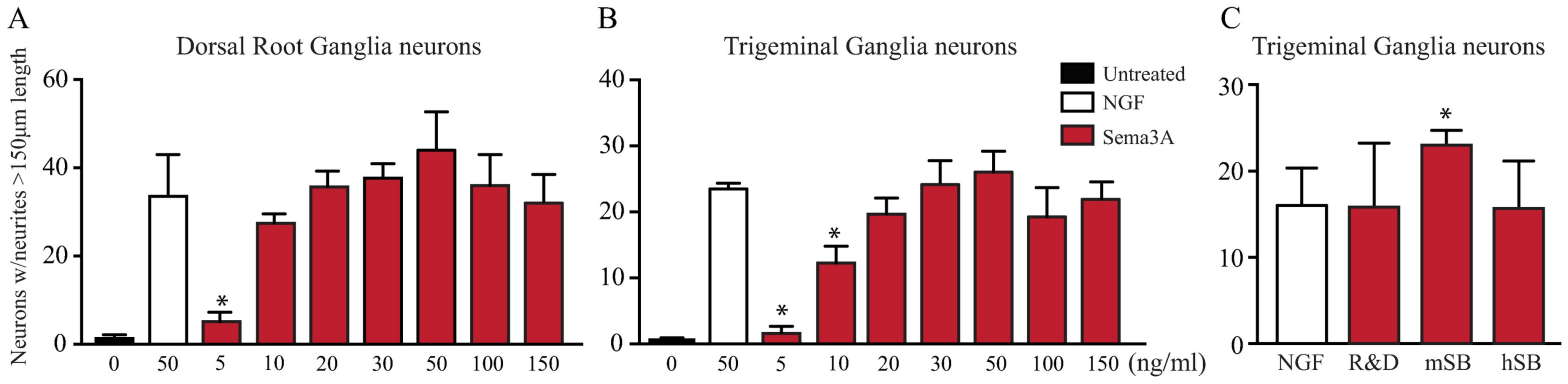
Sema3A is a potent inducer of neuronal growth. Since Sema3A is highly expressed in the cornea upon injury and does not inhibit the NGF induced growth on adult PNS neurons, we evaluated the effects of adding Sema3A alone to cultured neurons and compared to the NGF induced growth. **(A)** As described above DRG neurons responded well to NGF treatment. Surprisingly, Sema3A by itself is also a potent inducer of neuronal growth and similar neurite extension was observed at doses of 20 ng/ml Sema3A or higher. **(B)** Similarly, Sema3A is also a potent inducer of neuronal growth on TG neurons, and the number of TG neurons showing neurite growth was similar to that of neurons treated with NGF alone when incubated with Sema3A at 10 ng/ml or higher. **(C)** Sema3A from different sources, recombinant human or mouse, induced equally strong neuronal growth of TG neurons at equal concentrations. Values represent mean ± SD, experiments were performed in triplicate, each dish had an average of 200 neurons for DRG and 60 neurons for TG, and all neurons in a dish were evaluated. Neurite growth was evaluated at day 4 for DRG neurons and at day 3 for TG neurons. * indicate p<0.05 vs NGF. (R&D = from R&D Systems, m = murine, h = human, SB = Sino Biological Inc.).

**Fig 5:**
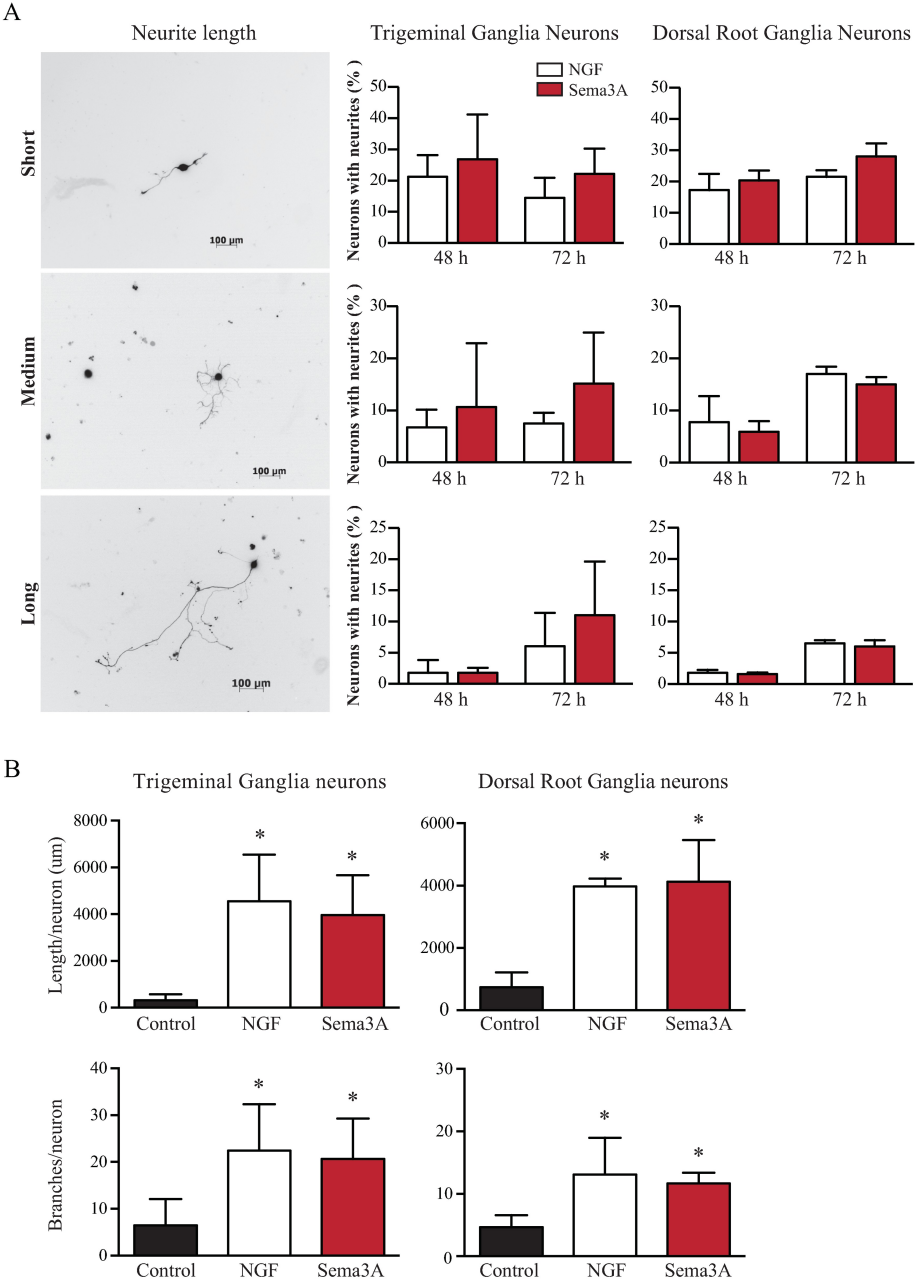
Sema3A induction of neuronal growth is comparable to NGF. The neuronal promoting effects of Sema3A and NGF were compared side by side in adult TG and DRG neurons. Neurons were treated either with growth medium alone as negative control or with 50ng/ml NGF or Sema3A. **(A)** The length of the neurites was quantified after 48 and 72 h post treatment and expressed as percentage of the total neurons in the dish. In TG neurons, Sema3A induced higher percentage of cells with neurites, however quantification shows that this effect was not statistically significant. In DRG neurons the percentage of short, medium or long neurites was comparable between Sema3A and NGF treatment. **(B)** The average length and number of branches of long neurites was compared and significant differences observed when compared to untreated control neurons, but similar effects observed between Sema3A and NGF treatments. Values represent mean ± SD, experiments were performed in triplicate, each dish had an average of 200 neurons for DRG and 60 neurons for TG; all neurons in every dish were evaluated. * indicates p<0.05 vs Control.

Since Sema3A is highly expressed in the cornea but does not affect NGF-induced neuronal growth of adult PNS derived neurons, we set out to determine its functions by treating adult PNS neurons with Sema3A alone. Surprisingly, we observed that Sema3A induced strong neuronal growth in DRG neurons (Fig 4A) as well as in TG neurons (Fig 4B) at 20 ng/ml and above, with highest neurite induction at 50ng/ml. Interestingly, the neuronal growth observed in both type of neurons when treated with Sema3A was similar to that observed in cells treated with NGF alone. We ruled out the possibility that these effects maybe due to a specific batch or brand of Sema3A by treating isolated TG neurons with Sema3A from different sources (human and mouse recombinant proteins from different providers). We found that neuronal growth was similar or better than NGF alone independently of the recombinant protein species or brand used (Fig 4C). When we compared side by side the growth promoting effects of Sema3A and NGF independently of each other, we found that both treatments induced similar responses in both DRG and TG neurons. The number of neurites per neurons was statistically similar regardless of the length of the neurites (Fig 5A) and the average length of long neurites and the number of branches was comparable between the treatments (Fig 5B). Our results identify Sema3A with potent growth promoting effects in PNS neurons, similar to that observed and described for NGF.

### Sema3A is a potent inducer of cornea nerve regeneration

**Fig 6:**
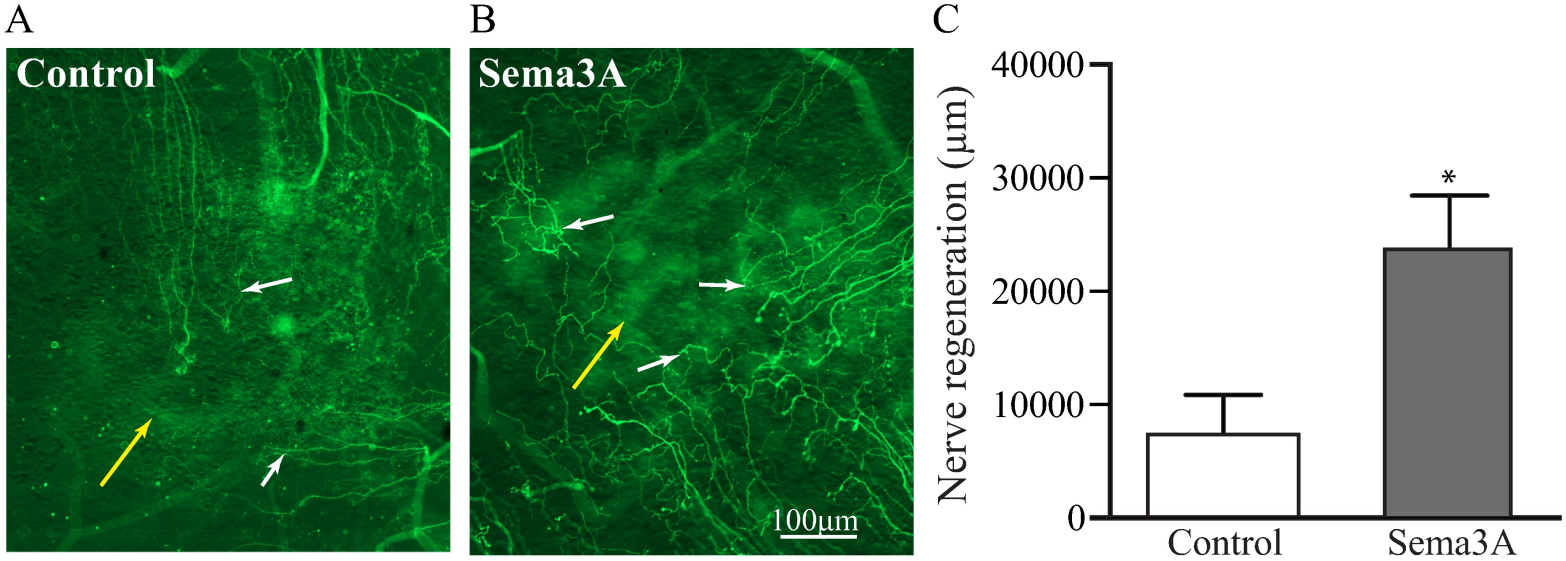
Sema3A induce nerve regeneration in injured corneas. The neuronal promoting effects of Sema3A were tested on thy1-YFP mice subjected to superficial corneal epithelial debridement. The debridement of the epithelium also removes the sub basal nerve plexus without affecting the cornea nerves in the stroma. Insertion of an intrastromal pellet containing Sema3A or vehicle (see Material and methods) allows for the slow release into the cornea. **(A**) Pellets containing vehicle (PBS) induced a discrete growth of sub basal nerves into the injured area. **(B)** However, addition of Sema3A induced faster regeneration of the superficial corneal nerves and higher nerve density was observed. **(C)** Quantification of nerve regeneration in the corneal injured area shows that Sema3A induced 3 folds higher nerve regeneration than control mice. White arrows = superficial nerves, yellow arrows = pellet. Values represent mean ± SEM, experiments were performed in triplicate, n = 5 per treatment, * indicates p<0.05 by ANOVA.

We performed in vivo studies to corroborate the neuronal growth effect of Sema3A on adult PNS derived neurons. For this thy1-YFP mice were subjected to corneal epithelium debridement to remove the central corneal epithelium and the superficial nerve plexus, leaving the corneal stroma intact. Immediately after this procedure, a pellet containing either Sema3A or vehicle (PBS) was inserted into the stromal micropocket as previously described [20]. Animals were observed daily and after 7 days the corneas were excised and stained for Sema3A expression and to visualize the regeneration of corneal nerves using β-3 tubulin staining. We observed no signs of corneal neovascularization, infection or inflammation in any of the mice tested. Sema3A expression was clearly visible in the corneal epithelium and in the inserted pellet loaded with Sema3A (S4 Fig). Nerve growth was evaluated on the total debrided area by analyzing mosaic images as described in Material and Methods. When we compared nerve regeneration, we found that control mice treated with vehicle showed some neuronal growth (Fig 6A). However, mice treated with Sema3A showed increased nerve growth extension and nerve density (Fig 6B). Images shown (in Fig 6A and 6B) are focused on the superficial nerves growing above the inserted pellet (white arrows). The presence of the pellet (yellow arrow) give the appearance of cloudiness but the cornea is transparent. Quantification of nerve regeneration expressed as total nerve length, was performed by tracing the superficial nerve fibers in the whole corneal debrided area using Neurolucida software. This analysis shows that Sema3A induced almost three fold higher nerve growth than vehicle (Fig 6C). In addition, Sema3A from either recombinant human or mouse sources were equally beneficial on inducing nerve regeneration.

## Discussion

The role of Sema3A in the cornea is still a matter of controversy. Previous studies have shown that Sema3A is highly expressed in the eye during mouse development and in the normal and injured adult cornea in rats [17, 18, 31]. In 1997 Tanelian *et al*.[8] used a gene gun plasmid transfection technique in rabbit cornea to demonstrate that SemaIII was able to inhibit the nerve regeneration from a corneal epithelium debridement model. These findings were in line with the well known axonal repulsion and inhibitory roles of Sema3 during development. Since then very few publications have followed this study, and fewer yet have explored its role in the eye. One study by Omoto *et al*. [32] provided evidence that an inhibitor of Sema3A accelerates nerve growth in a murine corneal transplantation model. Additional studies have shown that Sema3A induced axonal growth cone collapse in adult rat DRG using a signaling pathway distinct to that described in embryonic neurons [26]. This Sema3A inhibition of axonal growth in DRG neurons maybe restricted to neurons of small cell body diameter [33] and could function independently of growth cone repulsion [34]. However, other studies have shown a growth promoting effect of Sema3A in adult hippocampal neurons [12] and in the neuronal cell line PC12 [35].

Here, we used two models of PNS neurons to evaluate the effect of Sema3A especially in the cornea, and we found a 3 fold increase in Sema3A gene expression after corneal epithelium injury, which correlates well with the high expression levels documented during development, adulthood and post injury [17, 18, 31]. First, we confirmed that NGF is a strong inducer of neurite growth in both embryonic and adult DRG neurons. Secondly, we found that addition of Sema3A to embryonic neurons with well-developed axons and axonal growth cones resulted in axon retraction and growth cone collapse noticeable as early as 30 min post Sema3A application. However, in adult DRG neurons no inhibitory effect of Sema3A was observed and neurites showed elongation similar to cells treated with NGF alone. We further corroborated the lack of inhibitory effects of Sema3A in adult DRG by performing dose response experiments, in which neurons were treated with NGF and differential doses of Sema3A. Again no inhibition of NGF induced neurite elongation or branching was observed when adding Sema3A. Similar results were obtained when we treated TG neurons. Collectively, these results strongly suggested that in our model Sema3A plays opposing roles during development versus adulthood.

Surprisingly, we found that treatment of both DRG and TG isolated neurons with various concentrations of Sema3A alone matched the neuronal induction of cells treated with NGF. Thus, our data indicate that Sema3A is a strong inducer of neuronal growth and its lone effects were comparable to that induced by NGF alone. We also used recombinant human and mouse Sema3A from different sources and verified that this effect was not due to a technical phenomenon. Combined with the demonstrated increase in Semaphorin expression after corneal injury, these results suggested that Sema3A is a candidate molecule to promote neuroregeneration in vivo. This theory was further confirmed in our in vivo studies. PNS derived neurons from the TG that innervate the cornea were injured using the cornea epithelium debridement procedure. This technique allows for removal of the sub basal nerves without damaging the underlying stromal nerve bundles. After the injury, we inserted pellets containing Sema3A into an intrastromal micropocket, which allows the slow release of Sema3A into the cornea and the determination of its effect on nerve regeneration. Mice treated with Sema3A showed 3 fold higher increase in nerve regeneration than mice treated with vehicle, which showed discreet nerve regrowth. Additionally, we observed no angiogenic or inflammatory effects in any of the mice treated with Sema3A, an indication of the direct effect of Sema3A on nerve growth that resembles their effect observed in vitro.

These findings are in agreement with the discussed growth promoting effect of Sema3A on hippocampal and PC12 neurons and are the first to show that the high level of expression of Sema3A in the cornea may be related to its growth promoting effect. Our model of corneal nerve injury and delivery of Sema3A is different from the gene transfection in rabbit cornea [8], and we have used our model in our previous publications that demonstrate controllable and measurable quantification of nerve regeneration using transgenic neurofluorescent mice [3, 20, 21]. Our in vivo findings are strongly supported by the in vitro data and both showed that Sema3A induced neuronal growth in PNS neuronal models.

In summary, we have found that Sema3A has a potent growth-promoting role in adult PNS neurons, in contrast to its well-defined role as an axon repellent in embryos. How this switch in function from development to adulthood occurs is a matter of further studies, but it is not unusual to find dual roles for axon guidance proteins, as has been described for Netrins, Slits and Ephrin [6, 36]. Our study provides a better understanding of the role of the Sema3A in the mouse cornea, which in turn is important for designing the proper strategy to harness the effect of different growth factors for enhancing nerve repair.

## Supporting information

S1 Fig RT-PCR raw data. Gel images of Semaphorin expression in corneal and TG cells and tissues. These images were used to compile Fig 1A. (**A**) Expression of Sema3A, Sema3B, Sema3C, Sema3D and Sema3E. (**B**) Expression of Sema3F and RT minus control. (**C**) Expression of GAPDH. Lanes reads as follow: 1 = corneal cells, 2 = corneal tissue, 3 = TG neurons, 4 = TG tissue, 5 = Embryo tissue.
(TIF)

S2 Fig Phase images of embryonic and adult DRG neurons. Embryonic and adult neurons treated with NGF were images before (**A, C**) and after (**B, D**) addition of Sema3A. (**A** and **B**) 10x mosaic images showing DRG neurons with large neuronal processes, yellow dashed boxes indicate the cropped section used for Fig 2A and 2B. (C, D) Uncropped 10x images used for Fig 2C and 2D.
(TIF)

S3 Fig Video showing Sema3A induced growth cone collapse and axonal retraction. Embryonic DRG neurons were pretreated with NGF to induce neuronal growth. At day 3 Sema3A was added to the culture and its effects on neuronal growth was video recorded using time lapse imaging every 15 min. This video is focused on the neurons shown in Fig 2A and 2B.
(AVI)

S4 Fig Sema3A expression in corneas with intrastromal pellets. (**A**) Corneal whole mount of mice subjected to corneal epithelium debridement and intrastromal pellet implantation containing vehicle (**a-d**) or Sema3A (**e-h**). Phase images showing the presence of the implanted pellet (**a**, **e**). Rabbit anti Sema3A antibody (red) clearly stained the Sema3A filled pellet (**f**) but not the vehicle pellets (PBS) (**b**). Images focused on the cornea epithelium (**c**, **g**) and enlarged on **d** and **h**, clearly shows Sema3A expression in the cornea epithelium. (**B**) Cornea cryosections of mice subjected to cornea epithelium debridement only and mice subjected to corneal epithelium debridement plus pellet implantation. The expression of Sema3A (red) was clearly visible on the corneal epithelium as well as in the implanted pellet containing Sema3A. Yellow arrows = pellet, n = 4 animals.
(TIF)
