# Supplementary figures and images for: Semaphorin3A induces nerve regeneration in the adult cornea-a switch from its repulsive role in development
Source: PLoS One. 2018 Jan 25;13(1):e0191962. doi: 10.1371/journal.pone.0191962 (PMC5785010; doi:10.1371/journal.pone.0191962)

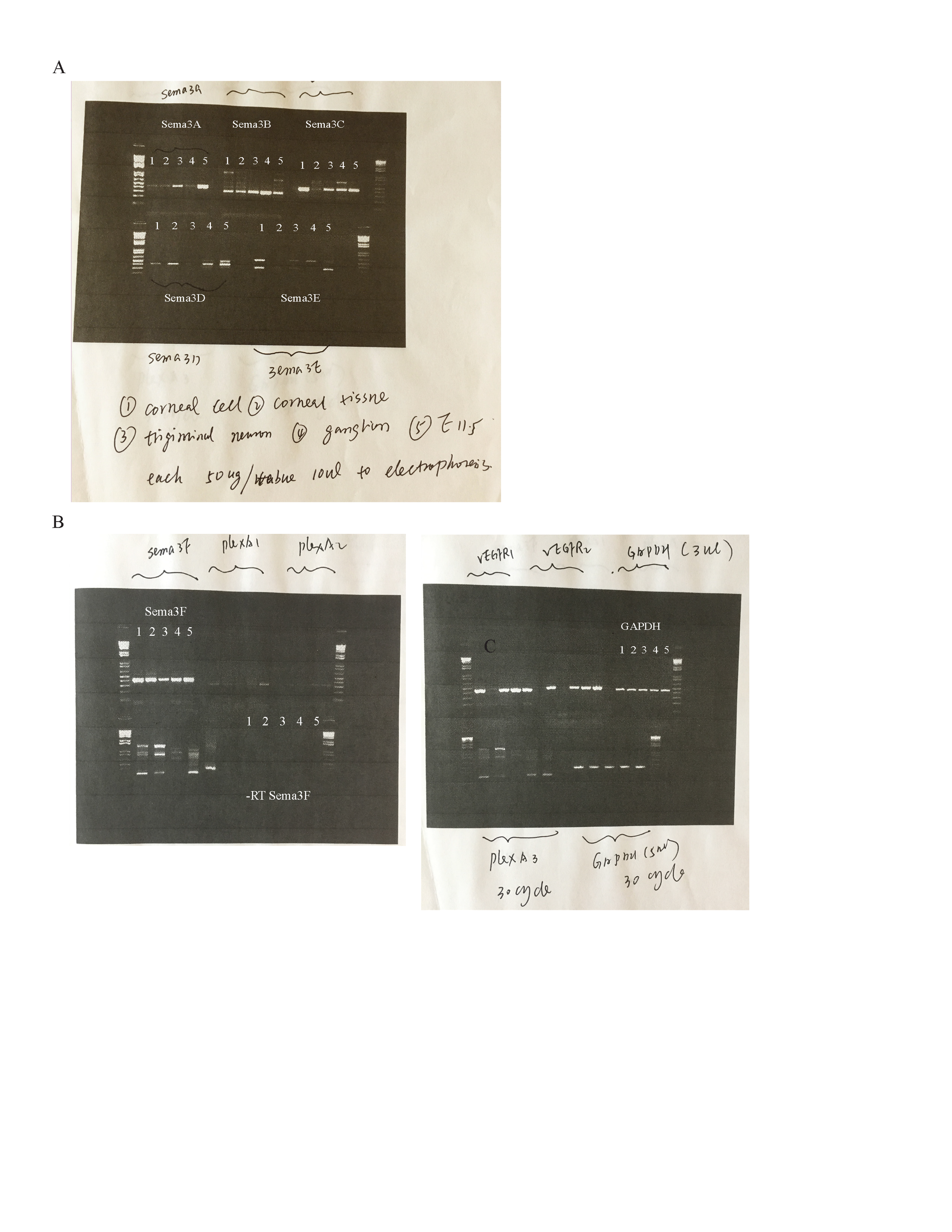

Supplement: S1 Fig — Gel images of Semaphorin expression in corneal and TG cells and tissues. These images were used to compile Fig 1A. (A) Expression of Sema3A, Sema3B, Sema3C, Sema3D and Sema3E. (B) Expression of Sema3F and RT minus control. (C) Expression of GAPDH. Lanes reads as follow: 1 = corneal cells, 2 = corneal tissue, 3 = TG neurons, 4 = TG tissue, 5 = Embryo tissue. (TIF) [file pone.0191962.s001.tif]

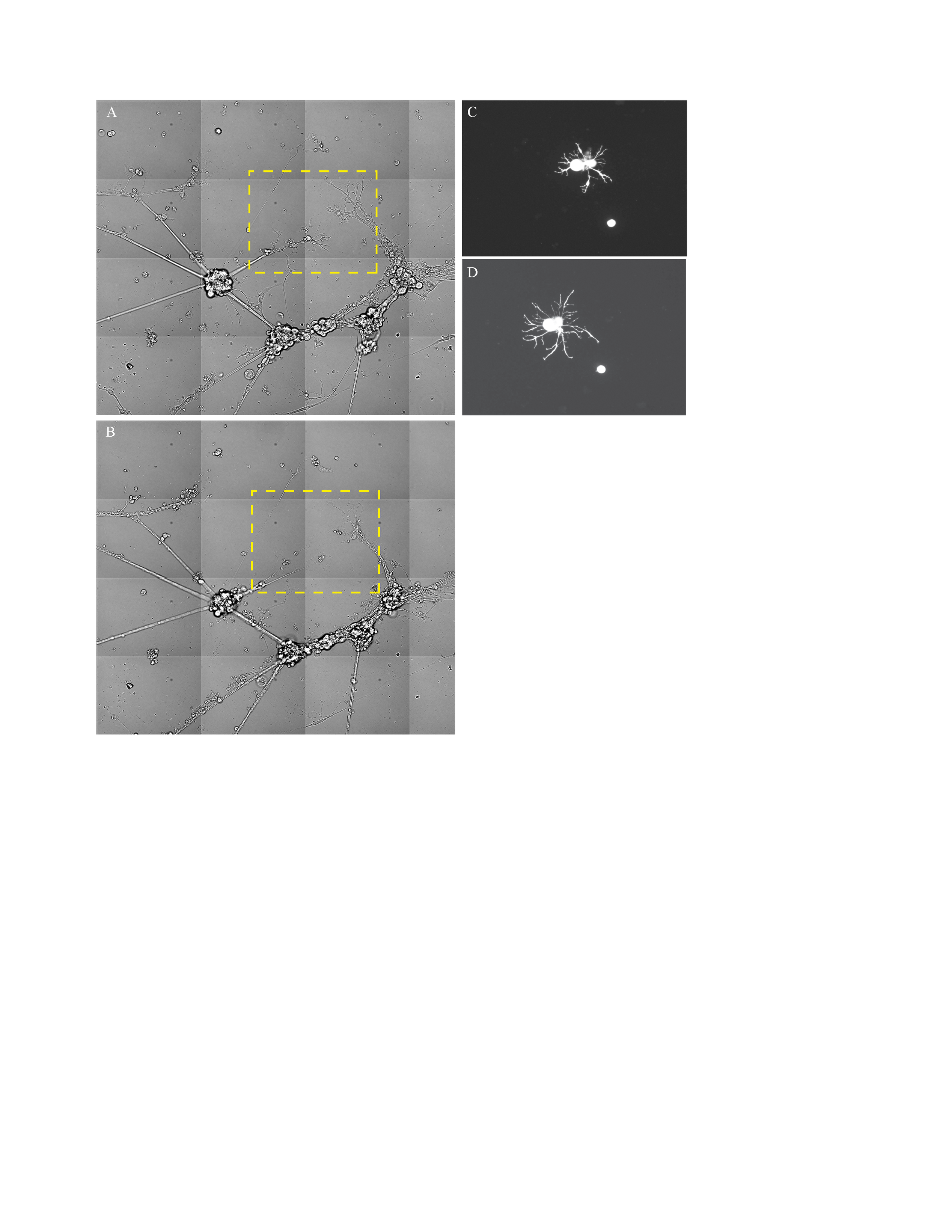

Supplement: S2 Fig — Embryonic and adult neurons treated with NGF were images before (A, C) and after (B, D) addition of Sema3A. (A and B) 10x mosaic images showing DRG neurons with large neuronal processes, yellow dashed boxes indicate the cropped section used for Fig 2A and 2B. (C, D) Uncropped 10x images used for Fig 2C and 2D. (TIF) [file pone.0191962.s002.tif]

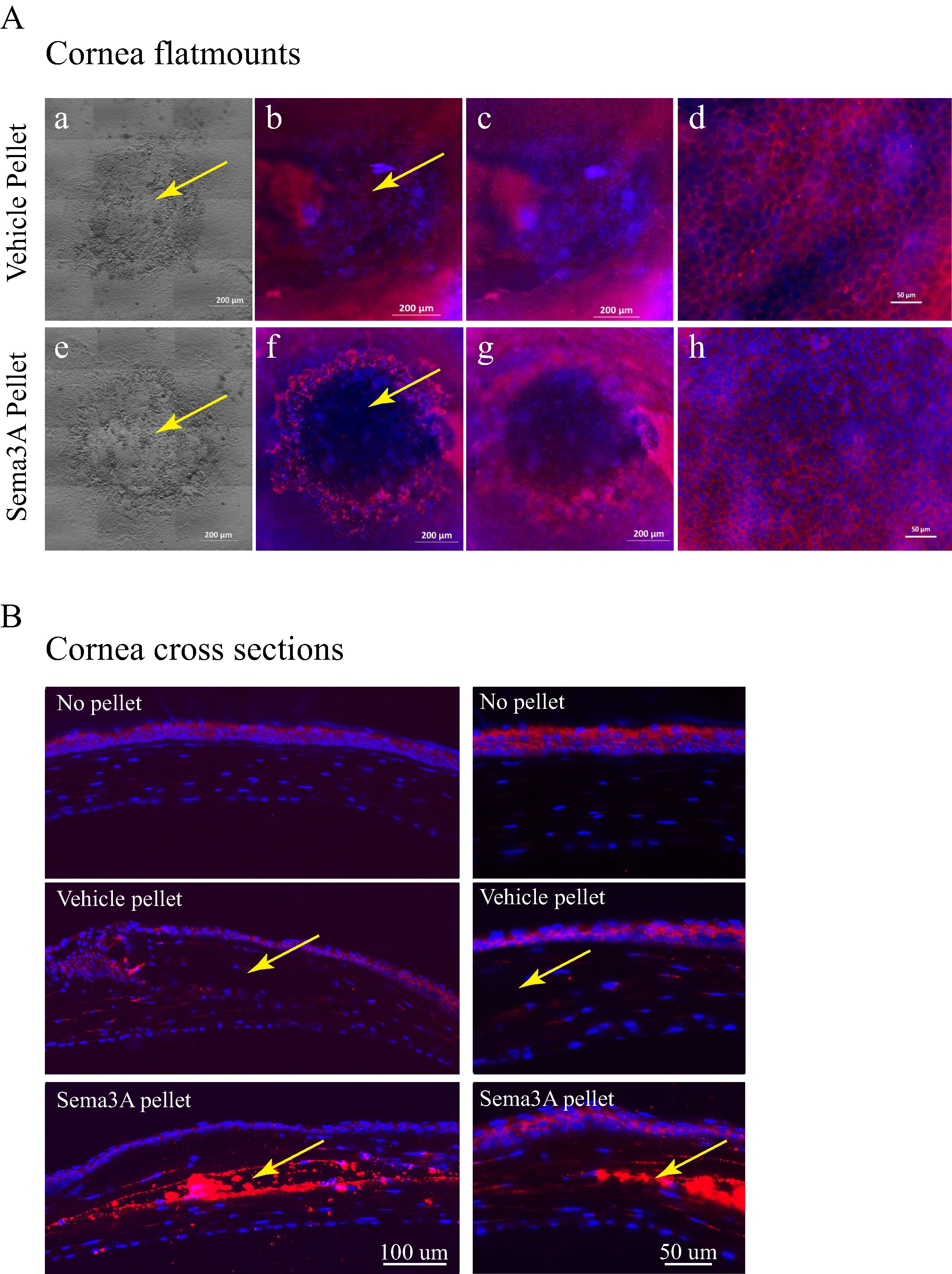

Supplement: S4 Fig — (A) Corneal whole mount of mice subjected to corneal epithelium debridement and intrastromal pellet implantation containing vehicle (a-d) or Sema3A (e-h). Phase images showing the presence of the implanted pellet (a, e). Rabbit anti Sema3A antibody (red) clearly stained the Sema3A filled pellet (f) but not the vehicle pellets (PBS) (b). Images focused on the cornea epithelium (c, g) and enlarged on d and h, clearly shows Sema3A expression in the cornea epithelium. (B) Cornea cryosections of mice subjected to cornea epithelium debridement only and mice subjected to corneal epithelium debridement plus pellet implantation. The expression of Sema3A (red) was clearly visible on the corneal epithelium as well as in the implanted pellet containing Sema3A. Yellow arrows = pellet, n = 4 animals. (TIF) [file pone.0191962.s004.tif]
